# Epidemiology and Risk Factor Analysis of Children with Bronchiolitis Admitted to the Intensive Care Unit at a Tertiary Care Center in Saudi Arabia

**DOI:** 10.3390/children10040646

**Published:** 2023-03-30

**Authors:** Sara Osman, Abdulqader Alaa adeen, Omar Hetta, Abdulaziz Alsiraihi, Mahmoud Bader, Alwaleed Aloufi, Amir Abushouk, Mohammed Yasir Al-hindi

**Affiliations:** 1 Department of Pediatrics, King Abdulaziz Medical City, Jeddah 22384, Saudi Arabia; alhindimo@ngha.med.sa; 2 Research Office, King Abdullah International Medical Research Centre, Ministry of National Guard Health Affairs, Jeddah 22384, Saudi Arabia; 3 College of Medicine, King Saud Bin Abdulaziz University for Health Science, Jeddah 22384, Saudi Arabia; 4 Department of Basic Medical Sciences, College of Medicine, King Saud Bin Abdulaziz University for Health Sciences, Jeddah 22384, Saudi Arabia

**Keywords:** acute bronchiolitis, pediatric intensive care, epidemiology, seasonality, risk factors

## Abstract

Bronchiolitis is a leading cause of hospitalization worldwide for children aged ≤2 years. Few studies have compared general ward and pediatric intensive care unit (PICU) admissions, particularly in Saudi Arabia. This retrospective cohort study aimed to compare the demographic and clinical characteristics of children with bronchiolitis admitted to the general ward with those admitted to the PICU. Children (≤6 years) previously diagnosed with bronchiolitis and admitted to the PICU or general ward at a tertiary center in Saudi Arabia between May 2016 and May 2021 were included. Multiplex polymerase chain reaction was used to identify respiratory viruses. Of the 417 patients enrolled, 67 (16.06%) were admitted to the PICU. The PICU group was younger (median, 2 months; interquartile range [IQR], 1–5 months) vs. (6 months; IQR, 2.65–13.25 months). There was a dramatic reduction in bronchiolitis admissions during the COVID-19 pandemic. The most common causative virus was respiratory syncytial virus (RSV) (54.9%). In the multivariate regression analysis, hypoxia, hyperinflation on X-ray, and non-RSV bronchiolitis were independently associated with PICU admission. However, a higher chronological age and cough were protective. Children with Down syndrome, immunodeficiency, or neuromuscular disorders, and intermediate preterm infants (29–33 weeks of gestation) are at a high risk of PICU admission (adjusted odds ratio: 2.4, 7.1, 2.9, and 2.9; *p* = 0.037, 0.046, 0.033, and 0.029, respectively). Bronchiolitis is still one of the leading causes of PICU admission. Particular attention should be paid to preventive measures, especially in the post-COVID-19 era, targeting high-risk groups.

## 1. Introduction

Bronchiolitis is the inflammation and obstruction of the bronchioles mainly caused by a viral infection in children younger than 2 years old. Additionally, it might extend to all parts of the airway in severe acute cases. Such a condition presents with difficulties in breathing, wheezing, fever, and cough. Respiratory syncytial virus (RSV) is a major etiological virus that causes annual bronchiolitis epidemics in infants [1]. Other causative pathogens are human-metapneumovirus, influenza, parainfluenza, and adenovirus. Bronchiolitis is considered to be a contagious disease that requires contact and droplet precautions [2].

Worldwide, bronchiolitis is the most common medical cause of hospitalization in children under 2 years old. Most reported cases occurred in developing countries, with higher rates of mortality attributed to malnutrition and insufficient supportive medical care [2]. Further research in Saudi Arabia reported that bronchiolitis is a seasonal disease that appears as an epidemic, particularly during winter [2]. Bronchiolitis requires public health attention because it affects approximately one-third of healthy children [3]. Of all cases, 2–10% need hospitalization, 5% of which require admission to the pediatric intensive care unit (PICU) [4,5]. The mortality burden of RSV infection in children was highlighted in the literature with a rate of 1 in 50 deaths in children aged 0–60 months and 1 in 28 deaths in children aged 28 days to 6 months [6]. Children who are less than 6 weeks of age, born prematurely, immunodeficient, or suffering from cardiopulmonary disorders are susceptible to severe bronchiolitis and might require passive immunization of palivizumab prophylaxis [2]. The need for mechanical ventilation or high-flow oxygenation is present in 2–3% of hospitalized children [5]. Despite efforts and high-quality guidelines, the number of admitted infants who have bronchiolitis continues to increase [2].

Literature comparing epidemiology and risk factors between general ward and PICU admission of children affected with acute bronchiolitis is scarce. A previous case–control study was conducted in the US covering admissions from 2015 to 2017 [7]. To the best of our knowledge, no study has been conducted in Saudi Arabia, the Middle East, or developing countries.

A better understanding of the clinical characteristics and epidemiological aspects of bronchiolitis will help to optimize healthcare delivery and introduce rapid intervention in this vulnerable population. To address this gap, this study was designed to compare the demographic and clinical characteristics of bronchiolitis among children admitted to the general ward with those among children admitted to the PICU.

## 2. Materials and Methods

This was a retrospective cohort study that analyzed the etiology and risk factors for bronchiolitis, resulting in admission to either the PICU (exposed) or the general ward (unexposed). This study was conducted at the general ward and PICU of the Pediatric Department of a tertiary care center between May 2016 and May 2021. The PICU is a tertiary unit with a 14-bed capacity, 5 of which are high-dependency beds, and an admission rate of approximately 550 patients annually. A nonprobability consecutive sampling technique was used. The inclusion criteria encompassed children younger than 6 years diagnosed with bronchiolitis and admitted to the PICU or general ward. The indications for general ward admission were high-risk patients, such as those with immune deficiency or congenital heart disease, hypoxia with oxygen saturation less than 90%, moderate to severe respiratory distress in the form of using accessory muscles, nasal flaring, or subcostal retractions, a decreased oral intake requiring intravenous rehydration, or significant x-ray findings indicating lung collapse, hyperinflation, or atelectasis. The indications for PICU admission were any patient that requires more than 5 L of oxygen therapy to maintain their oxygen saturation above 92%, moderate to severe respiratory distress that needs support with invasive or non-invasive ventilation, or abnormal blood gas with respiratory acidosis. Multiplex polymerase chain reaction was used to identify respiratory viruses. The multiplex used in our center detects 15 different viruses, including adenovirus, human metapneumovirus, human rhinovirus, influenza A and B, parainfluenza 1–4, RSV, and five different strains of coronavirus, including Middle East respiratory syndrome coronavirus. The sensitivity of RSV detection test that was used ranged from 80% to 90% for the age group included in the study. In addition, samples were collected via bronchoalveolar lavage or tracheal aspirate for patients who were on a ventilator, whereas samples were collected via nasopharyngeal aspirate for patients who were not on a ventilator. Patients suspected to have bronchiolitis with negative viral panel were diagnosed clinically. All patients discharged against medical advice were excluded from this study. Missing data were handled using pairwise deletion technique.

The BestCare 2.0 system (an electronic health medical record system) and the admission books of both the PICU and general ward were reviewed to retrospectively collect data. Approval with reference number IRBC/0740/21 was obtained before the initiation of the study.

The variables of interest included the age at the time of presentation, date of admission, gestational age, birth weight, leukocyte count, lymphocyte count, neutrophil count, hemoglobin, inflammatory markers (e.g., C-reactive protein [CRP] and procalcitonin), oxygen flow rate, oxygen saturation rate, heart rate, respiratory rate, length of stay in the general ward or PICU, the total length of hospital stay, duration of mechanical ventilation, duration of oxygen therapy, duration of antibiotic use, and duration of antiviral use. Additionally, categorical variables included sex, comorbidities, second-hand smoking, infecting organism, secondary bacterial infection, hyperinflation, peribronchial wall thickening on chest X-ray, PICU or general ward admission, type of management used, clinical features, complications, feeding method, and palivizumab immunoprophylaxis. Patient demographics, clinical presentation, and lab results were recorded upon admission. Management and complications were recorded throughout the course of admission.

Data were collected using Google Forms and extracted into a Microsoft Excel sheet. Statistical Package for the Social Sciences (SPSS), version 28, was used to analyze the data. For the purpose of the analysis, each admission was considered as one event. The admission rate per month was calculated. The descriptive statistics are presented as categorical and numerical variables. Categorical data were presented using frequencies, proportions, and bar charts. Numerical data were presented using frequency tables and histograms. Means and standard deviations were used for normally distributed data. Medians and interquartile ranges (IQRs) were used for skewed data. The inferential statistics are presented as categorical and numerical variables. The chi-square test or Fisher’s exact test was used to compare categorical data. For normally distributed numerical data, Student’s *t*-test was used. The Mann–Whitney U-test was used for numerical data with skewed distribution. Risk factors were analyzed using regression analysis. Two multivariate logistic regression models were developed to examine factors associated with PICU admission. The first model examined the clinical characteristics including sex, chronological age, hypoxia, cough, hyperinflation on X-ray, non-RSV-related bronchiolitis, and apnea. The second model examined the risk factors, including Down syndrome, immunodeficiency, gestational age categories, neuromuscular, congenital heart, and chronic lung diseases adjusted for age, sex, and non-RSV-related bronchiolitis. *p*-values of less than 0.05 were used to denote statistical significance, and all significant variables were analyzed to estimate the corresponding odds ratios and 95% confidence intervals.

## 3. Results

From May 2016 to May 2021, 762 patients were suspected of having bronchiolitis, of whom 331 patients (43.43%) were not included in the study as they were discharged from the emergency department as outpatients. Additionally, 14 patients (1.8%) were excluded as they were discharged against medical advice. Finally, 417 patients (54.72%) met the inclusion criteria and were enrolled in this study, of whom 176 were female (42.2%). Additionally, the data showed that the median birth weight was 2740 g (IQR, 2227.50–3250 g), the number of patients who were prematurely born was 103 (24.6%), and the median gestational age was 37 weeks (IQR, 36.25–39 weeks). The median chronological age was 5 months (IQR, 2–12 months). There were 350 patients (83.93%) admitted to the general ward compared to 67 patients (16.06%) who were admitted to PICU. In comparing the demographic characteristics between the general ward and PICU patients, the chronological age was significantly lower in PICU patients (Table 1).

**Figure 1:**
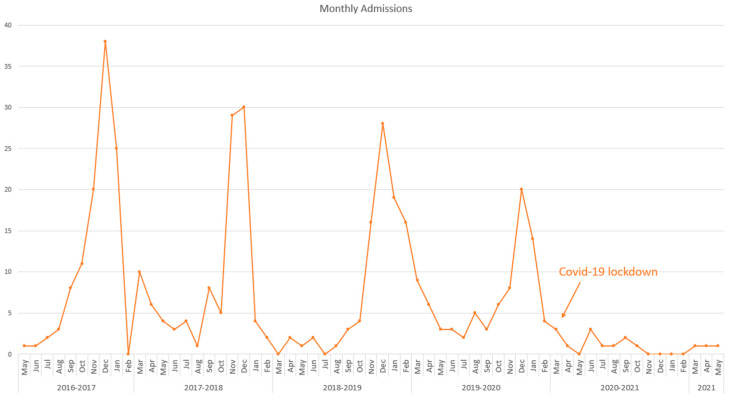
Admission numbers among bronchiolitis patients showed a seasonal pattern, with a spike in cases between September and March. However, during the coronavirus disease 2019 lockdown in March 2020, a remarkable decrease in admission was observed.

Admission numbers showed a seasonal pattern, with a spike in cases between September and March. However, during the coronavirus disease 2019 (COVID-19) lockdown in March 2020, a marked decrease in admission was observed (Figure 1). The main viruses recorded were RSV, with a slight increase in PICU patients compared with general ward patients (53.1% vs. 64.2%; *p* = 0.096); rhinovirus, with a significant increase among PICU patients (14.9% vs. 3.7%; *p* < 0.001); and influenza A, which was more prevalent in general ward patients than in PICU patients (3.4% vs. 3%; *p* = 1) (Table 2).

Generally, no significant differences in clinical characteristics were observed between the two groups in most categories. However, the prevalence of cough was significantly increased in the general ward group compared with that in the PICU group (90.9% vs. 82.1%; *p* = 0.032). Moreover, apnea (14.9% vs. 7.4%; *p* = 0.045), hypoxia (37.3% vs. 11.7%; *p* < 0.001), SpO2 (median, 94 vs. 96; *p* < 0.001), and lung hyperinflation on X-ray (47.8% vs. 31.7%; *p* = 0.011) were significantly increased in the PICU group compared with those in the general ward group. Regarding laboratory findings, a trend toward an increase in the median CRP levels was observed in the PICU group (Table 3).

The results of the multivariate regression analysis of clinical characteristics showed that non-RSV-related bronchiolitis, hyperinflation on X-ray, and hypoxia were significantly predictive of PICU admission; however, cough and a higher chronological age were significantly protective against PICU admission. Furthermore, the results of the multivariate regression analysis of the risk factors show that Down syndrome, immunodeficiency, and neuromuscular diseases were significant risk factors for PICU admission. Additionally, among all gestational age categories, the most susceptible group to be admitted to the PICU was the intermediate preterm gestational age category (Table 4).

Regarding management, the PICU group had an increase in bronchodilator use (89.6% vs. 79.7%; *p* = 0.059), nasal suctioning (91% vs. 58.3%; *p* < 0.001), and nasogastric (NG)/nasoduodenal (ND) feeding (82.1% vs. 8.6%; *p* < 0.001) compared with the general ward group. Similarly, the durations of antibiotics use (median, 5 days; IQR, 3–8 days vs. median, 2 days; IQR, 0–5 days; *p* < 0.001) and oxygen therapy (median, 4 days; IQR, 2–6 days vs. median, 0 days; IQR, 0–3 days; *p* < 0.001) were significantly longer among patients admitted to the PICU. As expected, continuous positive airway pressure therapy, high-flow nasal cannula, and mechanical ventilation were only used among PICU patients (range, 0–12, 0–4, 0–19 days, respectively). However, inhaled steroids were used more often in the general ward group than in the PICU group (26% vs. 14.9%; *p* = 0.053) (Table 4). The PICU group had a significantly longer hospital stay than the general ward group (median, 10 days; IQR, 7–13 days vs. median, 4 days; IQR, 3–7 days; *p* < 0.001). The organisms detected as secondary bacterial infection in both the general ward and the PICU included H. influenzae (6%), coagulase-negative Staphylococci (4.8%), E. coli (2.9%), *p*. aeruginosa (2.6%), and others, including S. aureus, K. pneumoniae, and S. pneumoniae. The PICU group also had a significantly higher prevalence of pneumonia than the general ward group (15% vs. 6.6%; *p* = 0.013) (Table 5). In our study, two patients died in the general ward, both of whom were in comfort care.

## 4. Discussion

Bronchiolitis is a substantial leading cause of childhood mortality and morbidity worldwide [8]. Despite the previous identification of risk factors, the possibility of immunization in high-risk groups, well-established guidelines, and the abundance of literature, bronchiolitis still poses a large public health burden [2,8].

In this study, among all patients included, PICU admission constituted 16.06%, which was lower than that documented by Richard et al. (published 2008) and Sala et al. (2015) (48.9% and 22%, respectively) [9,10]. However, in the study by Praznik et al. (2018), PICU admission constituted 3.85% of all bronchiolitis cases, which was lower than that in this study [11]. Such a trend toward a decrease in PICU admission could be explained by more centers around the world defining and following local or national guidelines with more clear indications for PICU admissions. This urges our center to follow a national guideline such as bronchiolitis in children: The Saudi initiative of bronchiolitis diagnosis, management, and prevention (SIBRO) [2].

The seasonal epidemic pattern of bronchiolitis admissions showed a remarkable increase in the period between September and March, with a spike of cases during December (Figure 1), which is in line with the literature [12,13]. During the COVID-19 era between November 2019 to February 2020 (pre-pandemic) and November 2020 to February 2021 (pandemic), the number of bronchiolitis hospitalizations declined dramatically. In line with our results, multiple publications in Brazil, North India, the US, and the United Kingdom reported identical reductions [14,15,16,17,18]. Additionally, a local study in Saudi Arabia reported a major reduction in bronchiolitis admissions by more than half in 2020 compared with that in the past 2 years, although it was statistically insignificant (*p* = 0.07) [19]. Such a reduction can be attributed to several factors, including the execution of precautionary measures, restriction of social gatherings, and national lockdown, which extremely affected the transmission dynamics of the disease.

As expected, RSV was the most prevalent virus causing bronchiolitis among all patients, resembling what has been documented in the literature [7,8,9]. Additionally, the prevalence rate of RSV infection in PICU patients was insignificantly higher than that in general ward patients. After regression analysis, non-RSV infection was an independent risk factor for PICU admission, supporting the literature that reported that patients with bronchiolitis infections caused by viruses other than RSV are more likely to be admitted to the PICU [20].

Similar to the literature, the results support that chronological age could be a predictor of the unit to where the patient would be admitted, which is due to the underdeveloped immune system of younger patients making them more likely to get infected [11]. With regard to the risk of prematurity, our findings showed that being intermediate preterm is a significant independent risk factor for PICU admission. The American Academy of Pediatrics (AAP) has updated its RSV prophylaxis guidelines five times since its inaugural license, most recently in 2019. Currently, the AAP recommendations for RSV prophylaxis are limited to premature infants of <29 weeks of gestational age (wGA) (previously <35 wGA), infants of <32 wGA with chronic lung disease, and infants < 12 months old with chronic heart disease. After the implementation of these guidelines, many published studies reported an increase in the rates of PICU admission and the use of mechanical ventilation among intermediate preterm patients, similar to our findings [21,22]. The new AAP restrictive guidelines might be concerning as this vulnerable age group has neither the capability to fight RSV nor the eligibility to be enrolled in the RSV prophylaxis program. In contrast to previous studies, median birth weight was an insignificant predictor of PICU admission [22].

In line with the literature, our analysis demonstrated that Down syndrome is an independent risk factor for PICU admission [23,24]. Children with Down syndrome are prone to severe respiratory diseases due to a decreased humoral and adaptive immunity [25]. The findings of regression analysis showed cough to be an independent protective factor against severe bronchiolitis infection. It is understood that infants, especially those that are less than 3 months old, are less likely to present with cough. However, the regression analysis shows cough to be an independent risk factor, which can be explained by the protective rule of coughing on the excretion of pathogens from the respiratory tract. PICU patients were significantly more likely to present with hypoxia and apnea. In line with our findings, an international study conducted at Tampere University Hospital in Finland between 2000 and 2015 reported that apnea is a significant risk factor for PICU admission [26]. On the other hand, other clinical presentations, such as tachypnea, cyanosis, wheezing, and fever were not associated with an increased risk of PICU admission. Therefore, the results of this study show an overlap in the clinical presentation of bronchiolitis between the two groups.

A study in King Abdulaziz Cardiac Center, Riyadh, Saudi Arabia, found that the hospitalization rate among patients who received palivizumab was 2.5%, which is consistent with the results of this study, proving the efficacy of palivizumab in preventing severe bronchiolitis necessitating PICU admission [27]. Unlike Tsou et al.’s study, the oxygen flow rate, heart rate, and hemoglobin level at admission showed no significant differences, whereas SpO2 levels at admission showed a significant difference [7]. The findings of our analysis show that hyperinflation on X-ray is an independent risk factor for PICU admission, similar to another cohort study that examined the X-ray findings of patients with bronchiolitis [28]. Aligned with a previous study, the median WBC count for both groups was not elevated [29]. Furthermore, the median CRP level was elevated in both groups, which is similar to that reported in a 2009 study [30].

The results of the multivariate analysis suggest that some clinical characteristics and risk factors were independently associated with high odds of severe bronchiolitis, leading to PICU admission. Historically, congenital lung diseases were proven to be associated with a more severe form of bronchiolitis [31]. However, our data showed that congenital lung diseases are not an independent predictor of PICU admission. The literature exploring such an association is contradicting [26]. Such a contradiction could be explained by the level of coverage of such infants with palivizumab: in the case of our center, the coverage is high, hence the offset of the association with the need for PICU admission [2]. In line with a systematic review, neuromuscular diseases were an independent significant risk factor for PICU admission due to bronchiolitis. Moreover, the literature states that immunosuppression is associated with a high mortality rate in children with RSV infections [32,33]. The analysis results support the literature documentation as they showed that immunodeficiency is an independent predictor of PICU admission.

In line with the literature, the use of bronchodilators and NG/ND feeding was higher among PICU patients, whereas the use of inhaled corticosteroids was higher among general ward patients [7]. Contrary to the literature, the use of systemic steroids showed no significant difference between PICU and general ward patients, which is due to updated guidelines being followed by our center [2,7]. Furthermore, the duration of antibiotic use was significantly higher in PICU patients, contradicting the study by Tsou et al. [7]. This could be due to the fact that our center is receiving more immunocompromised patients. PICU patients were significantly more likely to suffer from pneumonia as a complication. A prospective single-center study by Alejandre C. reported that the rate of pneumonia co-infection among their PICU patients with bronchiolitis was 15.7%, which relatively resembles the pneumonia co-infection rate documented among PICU patients in this study [34]. In line with the literature, the total length of hospital stay was longer among PICU patients [7].

The results of this study should be interpreted in the context of its strengths and limitations. Our study established the risk factors, clinical presentations, and epidemiology associated with PICU admission at the national level. Another strength is the revelation of the status of bronchiolitis admission during the pandemic and pre-pandemic periods of the COVID-19 era. However, this study has several limitations. First, as with other retrospective studies, this study was subjected to selection bias, which resulted in some missing data. Second, only common viral causes of bronchiolitis were recorded. However, there are other causative agents that were not recorded or tested for. Third, some nonmeasured variables might confound our results, such as socioeconomic status [35] and the number of siblings. Furthermore, some variables are stigmatized in society, such as household smoking and a lack of breastfeeding, which may yield inaccurate results. Fourth, the single-center nature of this study might limit the number of subjects and might under-represent the variety of socioeconomic and ethnic populations. It is recommended to have a long-term follow-up of infants discharged from the PICU to explore healthcare utilization and long-term detrimental effects [36].

## 5. Conclusions

Bronchiolitis is still one of the leading causes of PICU admission. Particular attention should be paid to preventive measures, especially in the post-COVID-19 era, targeting high-risk groups. National guidelines for diagnosis, management, and prevention should consider locally published data concerning seasonality and risk factor analyses. National multi-center active surveillance of acute lower respiratory tract illness is recommended.

## Figures and Tables

**Table 1:** Patient demographics.

	General Ward Admission (*n* = 350)	PICU Admission (*n* = 67)	*p*
Females, *n* (%)	146 (41.7)	30 (44.8)	0.642
Birth weight, median (IQR)	2736 (2274.25–3256)	2740 (2032.5–3202.50)	0.411
Preterm, *n* (%)	84 (24.3)	19 (28.8)	0.438
Gestational age, median (IQR)	37 (37–39)	37 (36–39)	0.683
Chronological age, median (IQR)	6 (2.75–13.25)	2 (1–5)	<0.001

PICU, pediatric intensive care unit; IQR, interquartile range.

**Table 2:** Causative agents of viral bronchiolitis in patients admitted to the general ward or PICU.

	General Ward Admission (*n* = 350)	PICU Admission (*n* = 67)	*p*
RSV, *n* (%)	186 (53.1)	43 (64.2)	0.096
Adenovirus, *n* (%)	2 (0.6)	1 (1.5)	0.41
Coronavirus, *n* (%)	1 (0.3)	1 (1.5)	0.296
Rhinovirus, *n* (%)	13 (3.7)	10 (14.9)	<0.001
Human metapneumovirus, *n* (%)	3 (0.9)	1 (1.5)	0.505
Influenza A, *n* (%)	12 (3.4)	2 (3)	1
Influenza B, *n* (%)	6 (1.7)	1 (1.5)	1
Parainfluenza virus, *n* (%)	3 (0.9)	1 (1.5)	0.505

PICU, pediatric intensive care unit; RSV, respiratory syncytial virus.

**Table 3:** Clinical features and lab investigations of patients according to admission status.

	General Ward Admission (*n* = 350)	PICU Admission (*n* = 67)	*p*
Down syndrome, *n* (%)	20 (5.7)	7(10.4)	0.149
Exposure to second-hand smoking, *n* (%)	15 (4.3)	2 (3.0)	0.662
Congenital respiratory anomalies, *n* (%)	19 (5.4)	4 (6.0)	0.859
Exclusive breastfeeding, *n* (%)	16 (6.6)	4 (9.1)	0.548
Following Palivizumab immunoprophylaxis program, *n* (%)	10 (2.9)	2 (3)	0.954
Tachypnea, *n* (%)	164 (46.9)	36 (53.7)	0.302
Dehydration, *n* (%)	48 (13.7)	11 (16.4)	0.561
Rhinorrhea, *n* (%)	163 (46.6)	26 (38.8)	0.242
Cyanosis, *n* (%)	60 (17.1)	15 (22.4)	0.306
Fever, *n* (%)	203 (58)	34 (50.7)	0.272
Wheezing, *n* (%)	125 (35.7)	23 (34.3)	0.828
Cough, *n* (%)	318 (90.9)	55 (82.1)	0.032
Crepitation, *n* (%)	132 (37.7)	22 (32.8)	0.488
Lethargy, *n* (%)	147 (42.0)	27 (40.3)	0.796
Apnea, *n* (%)	26 (7.4)	10 (14.9)	0.045
Hypoxia, *n* (%)	41 (11.7)	25 (37.3)	<0.001
Retraction, *n* (%)	145 (41.4)	33 (49.3)	0.235
SpO2, median (IQR)	96 (93–98)	94 (87–96)	<0.001
Oxygen flow rate, median (IQR)	1 (1–2)	2 (1–4.5)	0.378
Heart rate, median (IQR)	152 (139–168)	158 (140–173)	0.105
Respiratory rate, median (IQR)	48 (38–56)	50 (40–62)	0.212
Hyperinflation on X-ray, *n* (%)	111 (31.7)	32 (47.8)	0.011
Peribronchial wall infiltrates on X-ray, *n* (%)	259 (74.0)	55 (82.1)	0.16
WBC (10^3^/μL), median (IQR)	10.6 (7.7–13.47)	11.7 (7.85–15.35)	0.167
Neutrophil (10^3^/μL), median (IQR)	4.39 (2.33–6.39)	4.55 (2.79–6.78)	0.221
Lymphocyte (10^3^/μL), median (IQR)	4.16 (2.74–5.86)	4.59 (3.31–6.27)	0.313
Haemoglobin (g/dL), median (IQR)	11.5 (10.6–12.5)	11.4 (10.5–12.7)	0.916
C-Reactive Protein, median (IQR)	13.25 (3.5–35.42)	17.2 (5.25–53.25)	0.062
Procalcitonin, median (IQR)	0.13 (0.067–0.25)	0.3 (0.06–1.91)	0.323

PICU, pediatric intensive care unit; SD, standard deviation; SpO2, peripheral oxygen saturation; WBC, white blood cells; IQR, inter-quartile range.

**Table 4:** Risk factors predicting PICU admission.

	Unadjusted OR	Adjusted OR ^1^	*p*
Down syndrome	1.4	2.4	0.037
Neuromuscular	1.9	2.9	0.033
Significant CHD	0.26	0.33	0.30
Immunodeficiency	1.7	7.1	0.046
Chronic lung disease	1.2	1.9	0.24
Preterm < 29 wGA	0.73	0.84	0.8
GA categories			
Term (reference)	91 (26.0)	Reference	0.162
Late preterm (34–36 wGA)	258 (73.7)	0.901	0.809
Intermediate preterm (29–33 wGA)	9 (2.6)	2.931	0.029
Extreme preterm (<29 wGA)	320 (91.4)	0.929	0.911

GHD, congenital heart disease; GA, gestational age; wGA, weeks of gestational age; PICU, pediatric intensive care unit; OR, odds ratio. ^1^ Odds ratio of the association between the gestational age category and PICU admission was adjusted for the listed risk factors.

**Table 5:** Patient management and complications according to admission status.

	General Ward Admission (*n* = 350)	PICU Admission (*n* = 67)	*p*
Bronchodilators, *n* (%)	279 (79.7)	60 (89.6)	0.059
Oxygen therapy, median days (IQR)	0 (0–3)	4 (2–6)	<0.001
Mechanical ventilation, median days (IQR) *	0 (0–0)	0 (0–0)	<0.001
CPAP, median days (IQR)	0 (0–0)	3 (1–5)	<0.001
HFNC, median days (IQR) *	0 (0–0)	0 (0–0)	<0.001
Nasal suctioning, *n* (%)	204 (58.3)	61 (91.0)	<0.001
Anticholinergics, *n* (%)	23 (6.6)	1 (1.5)	0.149
Systemic steroids, *n* (%)	63 (18.0)	12 (17.9)	0.986
Inhaled steroids, *n* (%)	91 (26.0)	10 (14.9)	0.053
Hypertonic saline, *n* (%)	258 (73.7)	56 (83.6)	0.086
Anti-leukotriene, *n* (%)	9 (2.6)	1 (1.5)	1
IV fluid, *n* (%)	320 (91.4)	64 (95.5)	0.255
Antibiotics, median days (IQR)	2 (0–5)	5 (3–8)	<0.001
Antivirals, median days (IQR) *	0 (0–0)	0 (0–0)	0.839
NG/ND, *n* (%)	30 (8.7)	57 (80.3)	<0.001
Death, *n* (%)	2 (0.6)	0 (0)	1
TLOHS, median days (IQR)	4 (3–7)	10 (7–13)	<0.001
Secondary bacterial infection, *n* (%)	82 (23.4)	18 (26.9)	0.546
Pneumonia, *n* (%)	24 (6.9)	10 (14.9)	0.027

PICU, pediatric intensive care unit; SD, standard deviation; IQR, interquartile range; CPAP, continuous positive airway pressure; HFNC, high-flow nasal cannula; NG, nasogastric; ND, nasoduodenal. *: ranged from 25% to 75%; TLOHS, total length of hospital stay.

## Data Availability

The raw data supporting the results and conclusions of this article will be available by the authors upon request, without undue reservations.
